# Functional Imaging of Paragangliomas with an Emphasis on Von Hippel–Lindau-Associated Disease: A Mini Review

**DOI:** 10.15586/jkcvhl.2017.92

**Published:** 2017-09-04

**Authors:** Ioannis Ilias, Georgios Meristoudis

**Affiliations:** 1 Endocrine Unit, Elena Venizelou Hospital, Athens, Greece; 2 Department of Nuclear Medicine, Hippokration Hospital, Thessaloniki, Greece

**Keywords:** imaging, paraganglioma, pheochromocytoma, radionuclide, von Hippel-Lindau

## Abstract

Few reports have presented data and results on functional (i.e., nuclear medicine) imaging of paragangliomas and pheochromocytomas (PGLs/PHEOs) for von Hippel–Lindau (VHL) patients. Nuclear medicine localization modalities for chromaffin tumors can be specific or nonspecific. Specific methods make use of the expression of the human norepinephrine transporter (hNET) and vesicular monoamine transporters (VMATs) by these tumors. These permit the use of radiolabeled ligands that enter the synthesis and storage pathway of catecholamines. Nonspecific methods are not related to the synthesis, uptake, or storage of catecholamines but make use of the tumors’ high glucose metabolism or expression of somatostatin receptors. Consensuses and guidelines suggest that metastatic and sporadic PHEOs/PGLs in VHL patients (as in patients with chromaffin tumors of yet unknown genotype) should be evaluated first with ^18^F-dihydroxyphenylalanine (^18^F-DOPA) positron emission tomography/computed tomography (PET/CT). The functional imaging of second choice is ^123^I-metaiodobenzylguanidine (^123^I-MIBG) for PHEOs in VHL patients. ^123^I-MIBG, ^68^Ga-DOTATATE/DOTATOC/DOTANOC PET/CT, or ^18^F-fluorodeoxyglucose (^18^F-FDG) PET/CT can be a second choice of functional imaging for PGLs in VHL patients.

## Introduction

Pheochromocytomas and paragangliomas (PHEOs/PGLs; 70 and 30% of tumors, respectively) are rare chromaffin-cell neuroendocrine tumors represented by PGLs (developed from paraganglia which can be localized from the base of the skull to the pelvic floor) and PHEOs (or adrenal paragangliomas) (1, 2). Intra-adrenal PGLs are termed PHEOs (3); this is according to the World Health Organization’s most recent printed endocrine tumor classification dating to 2004 (4). This classification is still in effect (as a matter of fact initially, in the print version, benign intra-adrenal sympathetic PGLs were considered to be benign PHEOs (4), whereas more recently all intra-adrenal PGLs are termed PHEOs (5–8)). Thus, the terms PHEOs and PGLs can be used according to localization to define adrenal and extra-adrenal disease, respectively, or otherwise most neural crest-derived chromaffin tissue tumors can be acceptably termed PGLs (3). Hereditary forms account for 30% of cases (9). The prevalence of PHEOs is approximately 1/500,000 and that of PGLs is approximately 1/1,000,000. The incidence of von Hippel–Lindau (VHL) syndrome is estimated to be 1 in 36,000 births and the prevalence is estimated at 1/53,000 (10, 11). More than 500 inherited mutations in the VHL gene have been identified in people with VHL syndrome. Most often these are missense mutations in the VHL tumor suppressor gene (usually in codon 167; 3p25–26). Subjects with VHL develop hemangioblastomas, renal and pancreatic cysts, clear cell renal carcinomas, and pancreatic neuroendocrine tumors. Furthermore, approximately 25–50% of subjects with VHL syndrome have mostly benign PHEO/PGL (less than 5–15% are malignant, and slightly less than half of the patients show bilateral adrenal disease) (10, 12); they can also have sympathetic and head and neck PGLs (1). Generally, PGLs secret catecholamines or they can be nonsecreting, whereas PHEOs generally secrete catecholamines (2). Secreting PGLs (which are associated with the sympathetic system) are mainly thoraco-abdominopelvic. Patients with VHL and PHEOs/PGLs are considered to secrete predominantly norepinephrine (1). Nonsecreting PGLs are localized at the head and neck, and can manifest as asymptomatic masses or with symptoms associated with encroachment of nearby structures. No validated malignancy marker exists for PGLs (regarding about 15% of cases) with the exception of presumed or validated metastases. Diagnosis is based on clinical examination and family history. Young age at diagnosis and presence of multiple, extra-adrenal, bilateral adrenal, or malignant tumors are in favor of a hereditary form. Some authors recommend that any patient with a diagnosis of PHEO/PGL can benefit from genetic counseling (2). Others suggest a more restrictive counseling: in the case of extra-adrenal localization (PGL), bilateral PHEO, unilateral PHEO, and a family history positive for PHEO/PGL, and in any patient under 40 years with unilateral PHEO. The mutation research will be oriented according to the clinic, biology, and location of the tumor.

The first diagnostic step for PHEO/PGL involves biochemical testing for metanephrines and normetanephrines in blood or 24-h urine collections. These two tests have a good sensitivity >95% with a somewhat lower specificity of about 90–95% (2). Initial tumor localization is based on anatomical imaging, with computed tomography (CT) or magnetic resonance imaging (MRI). The typical appearance of PHEO/PGL is that of a spherical or ovoid lesion, with well-delimited tissue, with a certain heterogeneity, necrotic zones, and calcifications. The administration of a contrast agent according to the type of imaging helps to characterize the lesion. Due to the predominant intra-abdominal location of PHEO/PGL, abdominal and pelvic CT/MRI are the first-choice imaging modalities. The latter is recommended for patients with metastatic PGL and patients who have a contraindication of exposure to radiation (pregnant women, age below 25, etc.) (2, 13). Nevertheless, anatomical imaging, particularly for extra-adrenal disease, has shortcomings and further evaluation is warranted (14).

Further localization evaluation may require functional exploration by scintigraphy (i.e., nuclear medicine modalities) or positron emission tomography (PET) scan. Functional imaging is recommended in the baseline evaluation of patients with large PHEOs or PGLs, because size is linked with the risk of metastatic disease. Genetics also guides the implementation of functional imaging in the case of patients with syndromes known to harbor hereditary/bilateral/metastatic/malignant disease. Scintigraphy with ^123^I-metaiodobenzylguanidine (^123^I-MIBG) remains an examination of choice in this indication. On the other hand, Fluorine-18 (^18^F)-labeled fluorodeoxyglucose (^18^F-FDG) PET/CT would be superior to MIBG scintigraphy in the case of a known metastatic tumor.

Few reports have presented data and results of functional imaging on PGLs/PHEOs for VHL patients (Table 1). Herein, we briefly review the radiopharmaceuticals that have been used clinically for functional imaging on PGLs/PHEOs with an emphasis on VHL-associated disease.

**Table 1:** Selected published reports evaluating functional imaging of PHEO/PGL in VHL (only papers with at least five VHL patients were included)

Report	Radiopharmaceutical	*n* of VHL patients	Results/remarks	Accuracy of localization in bilateral adrenal disease
Srirangalingam et al. (32)	MIBG^a^	12	Overall accuracy of localization: 92%	40%
Rischke et al. (35)	^18^F-DOPA	19 (with multiple disease foci *n* = 6, with metastatic disease *n* = 1)	Sensitivity: On a per-patient basis: 89% On a per-lesion basis: 89%	–
Kaji et al. (37)	^18^F-FDA	7 (with bilateral adrenal disease *n* = 2)	Overall accuracy of localization: 100%	100%
	^123/131^I-MIBG		Overall accuracy of localization: 57%	100%
Weisbrod et al. (40)	^18^F-DOPA^b^	52 (with extrapancreatic disease *n* = 15)	Adrenal disease *n* = 10, bilateral adrenal disease *n* = 1, neck PGL *n* = 1	–
Taïeb et al. (15)	^131^I-MIBG	5 (with bilateral adrenal disease *n* = 3, extra-adrenal disease *n* = 1)	Sensitivity: On a per-patient basis: 60% On a per-lesion basis: 75%	100%

a In the report there is no distinction between ^123^I-MIBG and ^131^I-MIBG.

b Subjects were also studied with ^18^F-FDG PET but no details of imaging results or comparisons of imaging modalities’ results were given in the publication.

## Functional imaging in patients with PHEO/PGL

Nuclear medicine localization modalities for chromaffin tumors can be specific or non-specific. Specific methods make use of the expression of the human norepinephrine transporter (hNET) and vesicular monoamine transporters (VMATs) by these tumors (13). These transporters permit the use of radiolabeled ligands that enter the synthesis and storage pathway of catecholamines. Nonspecific methods are not related to the synthesis, uptake, or storage of catecholamines but make use of the tumors’ high glucose metabolism or expression of somatostatin receptors. Ideally, specific functional imaging methods should be used first and, if negative, nonspecific modalities should then follow, particularly if recurrent, metastatic, or malignant disease is suspected (13).

### Chromaffin-tumor-specific functional imaging

Meta-iodobenzylguanidine (MIBG) is a catecholamine precursor that is uptaken by chromaffin cells via hNET. Previous studies used iodine-131 (^131^I) MIBG, which was not very helpful in delineating metastatic or extra-adrenal PHEO/PGL (15, 16). Nowadays, it is labeled with iodine-123 (^123^I). It has no beta particle emission and its radiation exposure is low. Its half life is short (13.2 h) and allows higher doses to be injected; its principal emission photon energy (159 keV) lies closer to the 140 keV level (that of ^99m^Tc) around which gamma cameras are made to operate (at these energies the detection efficiency of scintillation crystal is satisfactory). ^123^I-MIBG permits tomographic imaging (single photon emission computed tomography (SPECT)). This allows the identification of small lesions that may not be evident on planar images. In addition, the combination of anatomical and functional information by hybrid SPECT/CT and SPECT/MRI imaging may increase the diagnostic accuracy. The sensitivity of ^123^I-MIBG is 85–88% and 56–76% and specificity is 70–100% and 84–100% for chromaffin tumors limited to the adrenals or for extra-adrenal localizations, respectively (17, 18).

Short-lived radioligands are used in PET, permitting functional imaging (including tomographic views) with higher spatial resolution than that delivered by conventional scintigraphic imaging. Dopamine is a catecholamine precursor and PET with ^18^F- fluorodopamine (^18^F-FDA) has been used with success in imaging adrenal and/or benign PHEOs or metastatic PHEOs/PGLs. The 110-min half-life of ^18^F permits tracers radiolabeled with this nuclide to be distributed to centers for diagnostic imaging that do not have on-site cyclotrons for preparing radiopharmaceuticals. Dihydroxyphenylalanine (DOPA) is converted into dopamine and then transported into PHEOs/PGLs via the large-type amino acid transporters (mainly LAT-1 and LAT-2) (19). PET with ^18^F-DOPA has been used for localizing PHEOs (sensitivity is reported at 89–97%) and is considered to be very accurate in imaging sporadic disease (19, 20). An advantage of ^18^F-DOPA PET over other modalities is that it does not show high uptake in normal adrenal glands (21).

### Non-chromaffin-tumor-specific functional imaging

**Figure 1:**
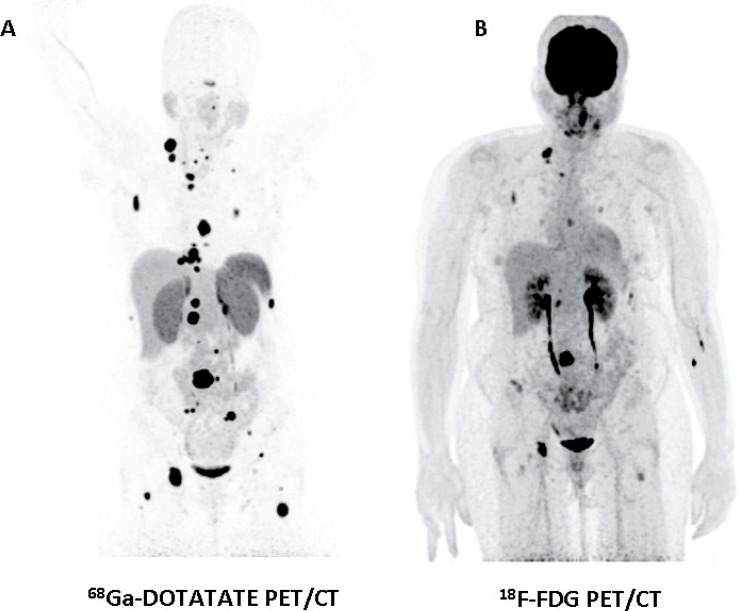
Anterior maximum intensity projection images of ^68^Ga-DOTATATE (A) and ^18^F-FDG (B) PET/CT of a 48-year-old female patient with metastatic PGL; note more foci of uptake with ^68^Ga-DOTATATE compared to ^18^F-FDG. (Images courtesy of Karel Pacak, MD, PhD, DSc, Section on Medical Neuroendocrinology, NICHD, NIH, Bethesda, MD, USA.)

Functional imaging with PET using ^18^F-FDG is currently widely available. This is a versatile modality that can localize various tumors and aid the staging of neoplastic disease. ^18^F-FDG PET is a convenient and accessible modality for localizing PHEOs/PGLs that are negative with specific functional imaging modalities (particularly metastatic disease) (Figure 1) (22). In patients with succinate dehydrogenase beta (SDHB)-associated PHEO/PGL—which are more prone to malignant disease—^18^F-FDG PET has 97–100% sensitivity in localizing tumor lesions, whereas the sensitivity of ^123^I-MIBG is 65–80% and that of ^18^F-FDA PET is 70–88% (23).

The expression of somatostatin receptors (ST-R) is common in PHEOs and PGLs (they mainly express type 2—mostly-, 3 and 5 ST-Rs (24); however, there are discrepancies in the literature and conflicting results have been presented). Octreotide is an octapeptidic somatostatin analog that is chelated with diethylenetriaminepentaacetate (DTPA) and labeled with indium-111 (^111^I-Pentetreotide) for ST-R scintigraphy (SRS). Although its splenic and renal accumulation is intense, it is nevertheless useful for localizing malignant/metastatic PHEOs/PGLs with a sensitivity approaching 90%. ST-R-based imaging may be less specific than ^18^F-DOPA PET imaging in the evaluation of PHEOs/PGLS (21). Furthermore, false positives may be expected in metastatic lymph nodes, meningiomas, and inflammation foci (21). Recently, novel 68 Gallium (^68^Ga)-DOTA-labeled somatostatin analogs (^68^Ga-DOTATATE, ^68^Ga-DOTANOC, and ^68^Ga-DOTATOC) have been introduced to clinical PET/CT use; they are reported to have higher sensitivity for detecting neuroendocrine tumors compared to “classic” SRS (25) (Figure 1). ^68^Ga-DOTATATE PET/CT was better than ^18^F-DOPA, ^18^F-FDA PET/CT, CT/MRI, and especially ^18^F-FDG PET/CT in imaging patients (*n* = 22) with sporadic metastatic PHEO/PGL (26), or head and neck PGLs (sensitivity reported at 93% in a series of 30 patients) (20).

## Functional imaging in patients with VHL

Although the literature is rich on the functional imaging of PHEOs/PGLs, few reports have presented data and results on VHL patients, and the numbers included were small.

The use of MIBG scintigraphy in a woman with VHL has changed fundamentally her management, as presented in an older case report, when scintigraphy indicated a metastatic PHEO in a lesion initially considered to be a brainstem hemangioblastoma (27); PHEO metastases were seen with ^123^I-MIBG in another case report (28). However, subjects with VHL have lower VMATs expression in chromaffin tissue, possibly hampering the use of MIBG as an effective functional imaging agent (29, 30). Additionally, ^123^I-MIBG uptake was not correlated with either hNET or VMAT (VMAT-1 in particular) expression in a series of 62 patients with PHEOs/PGLs, including two patients with VHL (31). In a comparison between VHL patients and SHDB patients, a study found that MIBG was overall positive in 11/12 VHL patients but was falsely negative in 3/5 VHL patients with synchronous bilateral adrenal PHEOs (32).

In a series of 116 patients with PHEOs/PGLs (and *n* = 3 with VHL), imaging with ^18^F-DOPA PET (lumped together in non-SDHx cases) had no false negative results (33). In an older series of 52 PHEOs/PGLs patients (and *n* = 2 with VHL, one with nonmetastatic and one with metastatic disease), PET with ^18^F-DOPA and ^18^F-FDA had showed the same lesions; both were better than ^123^I-MIBG in imaging the patient with metastatic disease (34). No influence of genotype on tumor ^18^F-DOPA uptake was noted in a PET–PET/CT study of 101 patients with PHEOs/PGLs; 19/101 were VHL patients (in one patient, ^18^F-DOPA was falsely negative, and sensitivity and specificity for the modality in VHL patients were calculated to be 89%) (35). Interestingly, in a study of 34 PHEO patients (of whom *n* = 3 reported with VHL), ^18^F-DOPA PET/CT had equivocal results for adrenal uptake (36). ^18^F-FDA PET was superior to ^123^I-MIBG in the context of VHL syndrome: in 7/7 VHL patients, all their PHEOs were imaged (two patients had bilateral disease) with the former modality, whereas the latter was positive in 4/7 patients (37).

PGLs in VHL patients are usually positive on ^18^F-FDG PET examinations (38); this positivity is probably more due to glucose transporters’ overexpression than increased glycolysis (38). ^18^F-FDG PET was positive in VHL patients with urinary bladder PGLs (*n* = 3) but no metastatic disease (39). In 52 VHL patients, ^18^F-FDG PET indicated the presence of more lesions than MRI or ^18^F-DOPA PET. It was superior for lesions in the pancreas and kidney; however, 30% of extrapancreatic lesions were seen only with ^18^F-DOPA PET and none of the other imaging techniques (40).

Since PHEOs/PGLs express to a high degree STRs type 2 (higher than 80%), ^68^Ga-DOTATATE PET/CT shows better diagnostic accuracy than “classic” SRS in the evaluation of PHEO patients; this can be attributed to the higher sensitivity of ^68^Ga-DOTATATE PET/CT (due to the high-quality images obtained with superior contrast and spatial resolution, and the higher affinity for STRs) (41). However, functional imaging with DOTA-compounds may be hampered by intense physiological uptake by the normal adrenal glands; small PHEOs or PGLs in VHL patients may be missed (20). In patients with VHL (*n* = 24) and gastrointestinal neuroendocrine tumors (but no reported PHEO/PGL), biomarkers for plasma tumors correlated with ^68^Ga-DOTATATE-calculated tumor volume (25). In a case report, ^68^Ga-DOTANOC PET/CT in a VHL patient detected both a cerebellar hemangioblastoma and a unilateral PHEO (42). In another case report, cerebellar lesions were found, but no PHEO (43).

Imaging with ST-R antagonists is an evolving domain: it has been used effectively for breast cancer (44) and pancreatic neuroendocrine tumors (45). In the future, they may be effectively used for PHEOs/PGLs, since apparently these ligands show higher tumor uptake compared to ST-R analogues and permit better tumor visualization (46–48). Unrelated to PHEOs/PGLs, a novel PET tracer, [89Zr]-bevacizumab (which binds VEGF-A) has been tried in VHL patients, visualizing hemangioblastomas (although these are benign nonmetastasizing tumors, if undetected they may lead to severe neurological deficits and death (49)), renal cell carcinomas, and brain metastases (50).

## Conclusion

Consensuses and guidelines suggest that metastatic and sporadic PHEOs/PGLs in VHL patients (as in patients with chromaffin tumors of yet unknown genotype) should be evaluated first with ^18^F-DOPA PET (21, 51). The functional imaging of second choice is ^123^I-MIBG for PHEOs in VHL patients. ^123^I-MIBG, ^68^Ga-DOTATATE/DOTATOC/DOTANOC PET/CT, or ^18^F-FDG PET/CT can be a second choice of functional imaging for PGLs in VHL patients (21). Additionally, in a very recent European Association of Nuclear Medicine guideline on the imaging of neuroendocrine tumors in general, including PGLs, preference is given to ^68^Ga-labelled SRS PET/CT over ^18^F-FDG PET/CT (52).

## Conflict of interest

The authors declare no potential conflicts of interest with respect to research, authorship, and/or publication of this article.
